# Anticancer Activity of Polyisoprenoids from *Avicennia alba *Blume. in WiDr Cells

**DOI:** 10.22037/ijpr.2019.1100719

**Published:** 2019

**Authors:** Didi Nurhadi Illian, Poppy Anjelisa Zaitun Hasibuan, Sumardi Sumardi, Arif Nuryawan, Ridha Wati, Mohammad Basyuni

**Affiliations:** a *Department of Pharmacology, Faculty of Pharmacy, University of Sumatera Utara, Medan, Indonesia.*; b *Department of Apothecary, Faculty of Pharmacy, University of Tjut Nyak Dhien, Medan, Indonesia. *; c *Department of Forestry, Faculty of Forestry, University of Sumatera Utara, Medan, Indonesia.*

**Keywords:** Polyisoprenoids, Avicennia alba, Apoptosis, Cell cycle, COX-2

## Abstract

Colorectal cancer is the third most common cancer world wide and has been occurred more in developing regions. The use of conventional chemotherapy agents may lead to various adverse effects. Therefore, it is required to find the potential drug for anticancer from alternative source of natural product including mangrove plants. The present study was conducted to determine the anticancer activity of polyisoprenoids from *Avicennia alba *Blume. leaves (PAL) in WiDr cells. Cell cycle inhibition, apoptosis activity, and suppression of cyclooxygenase-2 (COX-2) were also evaluated. The anticancer activity of PAL was determined by observing the activity of these compounds against WiDr cells using the [3-(4,5-dimetiltiazol-2-il)-2,5-difenil tetrazolium bromida] MTT assay. Inhibition of the cell cycle and increased apoptosis were analysed by flowcytometry. Suppression of COX-2 was analysed using immunocytochemistry. PAL exhibited anticancer activity against WiDr cells with an IC_50_ of 173.775 μg/mL. Cell cycle analysis revealed that the inhibition occurred in the G0-G1 phase, and apoptosis occurred in the early apoptosis phase. Furthermore, the result of an analysis of COX-2 expression showed that PAL enabled the suppression of COX-2 expression. PAL can be used as anticancer agents against WiDr colon cancer cells. However, *in-vivo* studies is required to confirm the *in-vitro* finding of the anticancer activity of polyisoprenoid extract.

## Introduction

Cancer is characterized by uncontrolled growth of a given cell type together with the invasion of surrounding tissue and spread to malignant cells (1). Cancer cells are recognized to lose the ability to down-regulate the cell cycle leading to their incessant proliferation (2). Apoptosis (program cell death) regulates the normal balance of cellular life and death which involve pro- and anti-apoptosis protein. Over expression of anti-apoptosis protein in the human cell is linked with cancer development, resistance in treatment and tumor progression (3). Cyclooxygenase-2 (COX-2), an inducible enzyme expressed at sites of inflammation, has recently emerged as a promising target for cancer therapy. Recent investigations have demonstrated that the over expression of COX-2 is correlated with progressive disease and poor prognosis, suggesting that COX-2 may play an important role in cancer development and metastasis, as well as suppresses apoptosis by altering the balance between anti-apoptotic and pro-apoptotic signals in cells (4, 5).

Colon cancer is the third most common cancer and the fourth leading cause of cancer death in the world (6). In Indonesia, 34,000 cases of colon cancer have been reported leading to the second most common cancer-causing death (7). Generally, the colon cancer incidence and mortality rates correlate with the adoption of a western lifestyle. However, they are still rising rapidly in many low-income and middle-income countries linked to ongoing societal and economic development, and, in highly developed countries, the rates are stabilizing or decreasing (8). The risk factor for colon cancer is also associated with healthy diets. Intakes of low fibre and high fat have increased the risk of colon cancer (9).

Primary cancer treatments have incorporated both chemotherapeutic agents and ionizing radiation to eliminate the bulk of the tumour mass. However, these treatments cause cancer relapse and even destroy healthy cells. The use of conventional cancer treatment also causes the development of drug resistance in tumour cells. Therefore, potential anticancer drugs need to be identified for anticancer from alternative sources, including natural products.

Mangroves are rich sources of secondary metabolites, mostly from isoprenoids compounds (10, 11). Identification of isoprenoids, as well as polyisoprenoids from the leaves and roots of the mangrove plant *Avicennia alba* (*A. alba*), has been described (12, 13). It has been reported that this plant possesses various pharmacological activities including anticancer and antiinflammatory (14). Polyisoprenoids showed some biological activities such as lowering cholesterol, antiinflammation, and antiulcer (15, 16). However, less information is available about the anticancer activity of polyisoprenoids from *A. alba*; therefore, the present study was aimed to investigate their anticancer activity. The effect of this extract on the cell cycle, apoptosis and suppression of COX-2 expression was also determined. The results of this study will help to characterize the anticancer properties of polyisoprenoids from *A. alba. *

## Experimental

*Plant and chemical materials*

Fresh leaves of *Avicennia alba* Blume. (Acanthaceae) were collected from Lubuk Kertang village, Langkat regency, North Sumatra province, Indonesia. The samples of plant material has been identified in the Research Centre for Oceanography, Indonesian Institute of Science, Jakarta, Indonesia and the voucher specimen has been deposited in the herbarium. Roswell Park Memorial Institute-1640 (RPMI-1640) and M199 were obtained from Gibco (Carlsbad, CA, USA), the Annexin-V and propidium iodide kits were purchased from BioLegend (San Diego, CA, USA), dimethylsulfoxide (DMSO), [3-(4,5-dimetiltiazol-2-il)-2,5-difenil tetrazolium bromida] (MTT) powder, phosphate-buffered saline (PBS), and sodium dodecyl sulphate (SDS) were purchased from Sigma–Aldrich (St. Louis, MO, USA). Chloroform, methanol, hexane, ethanol, HCl, and KOH were obtained from Merck (Darmstadt, Germany). The standardization of the extract has been previously described (13). The active polyisoprenoid compounds in the extract have been confirmed as dolichols family (C_60_–C_100_) (13).

*Preparation of polyisoprenoid extracts*

The procedure for the extraction of polyisoprenoids was performed as previously described (17-19). The *A. alba *leaves were dried at 60–75°C for 1–2 days. The dried leaves were crushed into a fine powder (200 g) and were immersed in 2000 mL of a mixture of chloroform:methanol (2:1, v/v) solvent for 48 h. The lipid ex­tract of the leaves was saponified at 65°C for 24 h in 86% ethanol containing 2 M KOH. The non-saponifiable lipids of leaves were extracted with hexane, and the organic solvent was evaporated and re-dissolved in hexane.

*Cytotoxic activity test*

Cytotoxic activity was determined using the MTT method with slight modifications (20). Briefly, WiDr cells (1 × 10^4 ^cells/well) were seeded and grown in a 96-well micro plate and were incubated for 24 h. Thereafter, the cells were treated with various concentrations of PAL (500, 250, 125, 62.5, 31.25 and 15.625 µg/mL, were performed as previously described) and were incubated 37°C in a 5% CO_2 _incubator for 24 h (21). Doxorubicin was used as a positive control. DMSO (1%) was used as the co solvent. After incubation, the culture media and test solutions were discarded, and then the cells were washed with PBS. Next, 100 µL of RPMI and 10 µL of MTT (5 mg/mL) were added into each well, and the cells were incubated for 4–6 h in a 5% CO_2 _incubator at a temperature of 37°C. The MTT reaction was stopped using a stopping reagent (10% SDS in 0.1 N HCl), and the cells were protected from light at room temperature and allowed to stand for one night. Cell viability was observed using an ELISA reader (BioTek EL 800, United States) at a wavelength of 595 nm. Living cells react with MTT to form a purple colour. The percentage viability was calculated using the following formula (22):

$\mathrm{Viability\%}(\mathrm{Living Cells})=\frac{\mathrm{Abs Treatment Cells}-\mathrm{Abs Media Control}}{\mathrm{Abs Media Control Cells}-\mathrm{Abs Media Control}}\times \mathrm{100\%}$

*Cell cycle inhibition analysis*

Cell cycle inhibition was assessed as described previously (23). Briefly, WiDr cells were platedin a 6-well microplate at adensity of approximately 5 × 10^5 ^cells/well and then were incubated for 24 h to obtain good growth. The next day, the cells were treated with various concentrations of PAL (1/2, 1/5 and 1/10 IC_50_, were carried out as previously reported) and were incubated for 24 h (21). Doxorubicin was used as a positive control. After incubation, the samples were transferred into 15-mL conical tubes, and the microplates were washed with PBS, which was then collected and added to the same conical tubes. Next, 250 µL of trypsin was added to the microplates, which were then incubated for 3 min at 37°C. Thereafter, 1 mL of culture media was added to the microplates, and then the media were collected and added to the same conical tubes. Next, 1 mL of PBS was added to the microplates, and then the PBS washes were collected and added to the same conical tubes, followed by centrifugation at 600 rpm for 5 min, and removal of the supernatants. Thereafter, the pellets were resuspended in 1 mL of PBS, followed by transfer to microtubes and centrifugation at 2000 rpm for 3 min. Next, 500 µL of 70% ethanol was added and incubated for 30 min. After incubation, the cells were resuspended in propidium iodide. The cell cycle distribution was observed using a flow cytometer (FACSCalibur).

*Apoptosis analysis*

Apoptosis detection was performed as described previously (23). Briefly, WiDr cells were grown in a 6-well microplate at a density of approximately 5 × 10^5 ^cells/wells and incubated for 24 h to obtain good growth. The following day, the cells were treated with various concentrations of PAL (1/2, 1/5, and 1/10 IC_50_) and incubated for 24 h. Doxorubicin was used as a positive control. After incubation, the samples were transferred into 15-mL conical tubes, the microplates were washed with PBS, and then the PBS washes were collected and added to the same conical tubes. Next, 250 µL of trypsin was added to the microplates, which were incubated for 3 min at 37°C. Thereafter, 1 mL of culture media was added to the microplates, and then the media were collected and added to the same conical tubes. Next, the cells in the microplates were resuspended in 1 mL PBS, the PBS washes were collected and added to the same conical tubes, followed by centrifugation at 600 rpm for 5 min and removal of the supernatants. The pellets were dissolved in 1 mL of PBS and were transferred into microtubes and centrifuged at 2000 rpm for 3 min. Next, 100 µL of Annexin-V buffer was added to the cells, followed by the addition of 5 µL each of Annexin-V and propidium iodide and incubation for 10 min. After incubation, 300 µL of Annexin-V buffer was added. Apoptosis was observed using a flow cytometer (FACSCalibur).

*Suppression of COX-2 expression*

Suppression of COX-2 was performed as described previously (24). Briefly, WiDr cells were grown on coverslips in24-well microplate at a density of 5 × 10^4 ^cells/well and were incubated for 24 h to obtain good growth. The following day, the cells were treated with various concentrations of PAL (1/2, 1/5, and 1/10 IC_50_) and were incubated for 24 h. Doxorubicin was used as a positive control (25). After incubation, the cells were washed with PBS. Next, cold methanol was added to the microplates, which were incubated in the freezer at –4°C for 10 min. After incubation, the methanol was removed, and the coverslips were placed on a dish. Next, the cells were washed with distilled water three times and were incubated with hydrogen peroxide blocking solution for 10 min at room temperature, followed by removal of the blocking solution. Next, the cells were incubated with prediluted blocking serum for 10 min at room temperature, followed by removal of the serum. After incubation, the COX-2 antibody was added, followed by incubation for 1 h at room temperature. After incubation, the coverslips were washed with PBS and then were incubated with secondary antibody (biotinylated universal secondary antibody) for 10 min. After incubation, the cells were washed with PBS and then were incubated with streptavidin-horse radish peroxidase enzyme and incubated for 10 min. Next, the cells were washed with PBS, DAB was added, and then the cells were incubated for 5 min (for brown colour development). After incubation, the cells were washed with PBS and distilled water, and then the cells were washed with Mayer-Haematoxylin solution and were incubated for 5 min. After incubation, the cells were washed with distilled water, followed by the addition of 70% ethanol and incubation for 2 min. Next, xylol solution was added to the cells, followed by drying. After drying, mounting media was added dropwise to the coverslips, which were covered with slides. Suppression of COX-2 was observed using a microscope with the optiLab system.

*Statistical analysis*

Data were represented as means ± SD from at least three independent experiments. The IC_50_ values was calculated from the linear regression equations of dose response curve for each experiment using probit analysis with SPSS 23 software. 

## Results and Discussion

*Cytotoxic activity analysis*

The search for potential anticancer from *A. alba* leaves exhibited the highest anticancer activity showing the lowest of IC_50 _value among eighteen mangrove plants (screening data not shown). Cytotoxic activity was carried out using the MTT method by measuring the intensity of colour (colourimetric) due to the metabolism of a substrate by living cells into a coloured product. In the present study, an MTT salt was used. This salt was involved in the action of the enzyme dehydrogenase. MTT is reduced to formazan by the succinate tetrazolium reductase system, which is included in the mitochondria of living cells (20). The viability effect of PAL against WiDr cells was exhibited in a dose-dependent manner in which a low concentration of extract (31.25 μg/mL) resulted in a high percentage of viability (99.881%). Conversely, the highest concentration (500 μg/mL) revealed a low percentage of viability (1.426%). As shown in Table 1, the IC_50_ value of PAL was 173.775 μg/mL, which considered as a potent agent. The results indicate that the polyisoprenoid extract from *A. alba *leaves was less active as an anticancer agent because of the extract is considered active if IC_50_ ≤ 100 μg/mL (26). However, it could still be developed as an anticancer agent because it is considered inactive if IC_50 _> 500 μg/mL (27). The PAL have been shown to contain 100% dolichols family (C_60_–C_100_), without any polyprenols detected (13). A previous study described that the methanol extracts of *A. alba* (stem bark and leaves) were active in causing anti-proliferative effects in both MCF-7 and T47D cell lines (28). In addition, this extract also exhibited cytotoxic properties against various human cancer cell lines including colon cancer cell line* e.g. *HT-29 (29). In case of pure compound, naphthoquinones isolated from dried aerial parts of *A. alba* had an anti-tumor-promoting activity in a short-term *in-vitro* assay of TPA-induced EBV-EA (Epstein–Barr virus) activation in Raji cells (30).

**Table 1:** IC50 of polyisoprenoid extract from *A. alba *and doxorubicin

**No.**	**Sample**	**IC** **50 ** **(μg/mL)**
1.	*Avicennia alba*	173.775
2.	Doxorubicin	15.550

**Table 2:** Distribution of WiDr cells after treatment with various concentrations of polyisoprenoid extract from *A. alba *leaves and doxorubicin (1/2, 1/5 and 1/10 IC50).

**Treatment**	**Concentration (μg/mL)**		**Cell Phase (%)**	
		**G0-G1**	**S**	**G2-M**
Control		72.20	15.83	11.23
Polyisoprenoid 1/2 IC50	86.88	62.62	17.72	15.29
Polyisoprenoid 1/5 IC50	34.77	66.10	20.35	11.75
Polyisoprenoid 1/10 IC50	17.38	70.01	16.46	12.77
Doxorubicin 1/2 IC50	7.77	14.21	52.32	31.18
Doxorubicin 1/5 IC50	3.11	62.87	18.57	17.42
Doxorubicin 1/10 IC50	1.56	61.91	21.66	16.34

**Table 3:** Apoptosis test results in WiDr cells after treatment with various concentrations of polyisoprenoid extract from *A. alba *leaves and doxorubicin (1/2, 1/5 and 1/10 IC50): R1 = live cells; R2 = early apoptotic cells; R3 = late apoptotic cells/early necrosis; R4 = late necrosis cells

**Treatment**	**Concentration (μg/mL)**	**(%)**			
		**R1**	**R2**	**R3**	**R4**
Control		96.27	1.43	2.02	0.30
Polyisoprenoid 1/2 IC50	86.88	42.24	25.85	22.85	9.22
Polyisoprenoid 1/5 IC50	34.77	57.89	32.66	7.66	2.15
Polyisoprenoid 1/10 IC50	17.38	88.27	6.44	3.76	1.62
Doxorubicin 1/2 IC50	7.77	16.89	0.57	0.41	82.23
Doxorubicin 1/5 IC50	3.11	51.63	3.26	0.67	45.08
Doxorubicin 1/10 IC50	1.56	78.29	3.55	3.62	14.85

**Figure 1:**
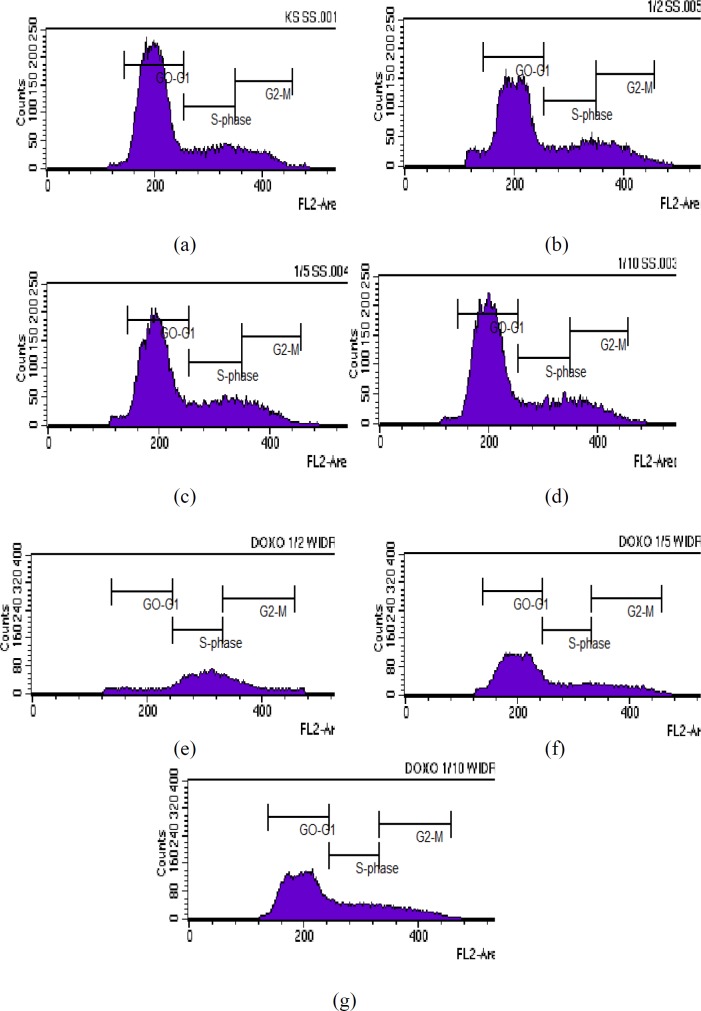
Results of cell cycle analysis in WiDr cells after treatment with various concentrations of polyisoprenoid (PI) extract from *A. alba *leaves and doxorubicin (1/2, 1/5 and 1/10 IC50): (a) control; (b) PI, 1/2 IC50; (c) PI, 1/5 IC50; (d) PI, 1/10 IC50; (e) doxorubicin, 1/2 IC50; (f) doxorubicin, 1/5 IC50; and (g) doxorubicin, 1/10 IC50. The known cytotoxic agents, doxorubicin has been tested as positive control (25).

**Figure 2:**
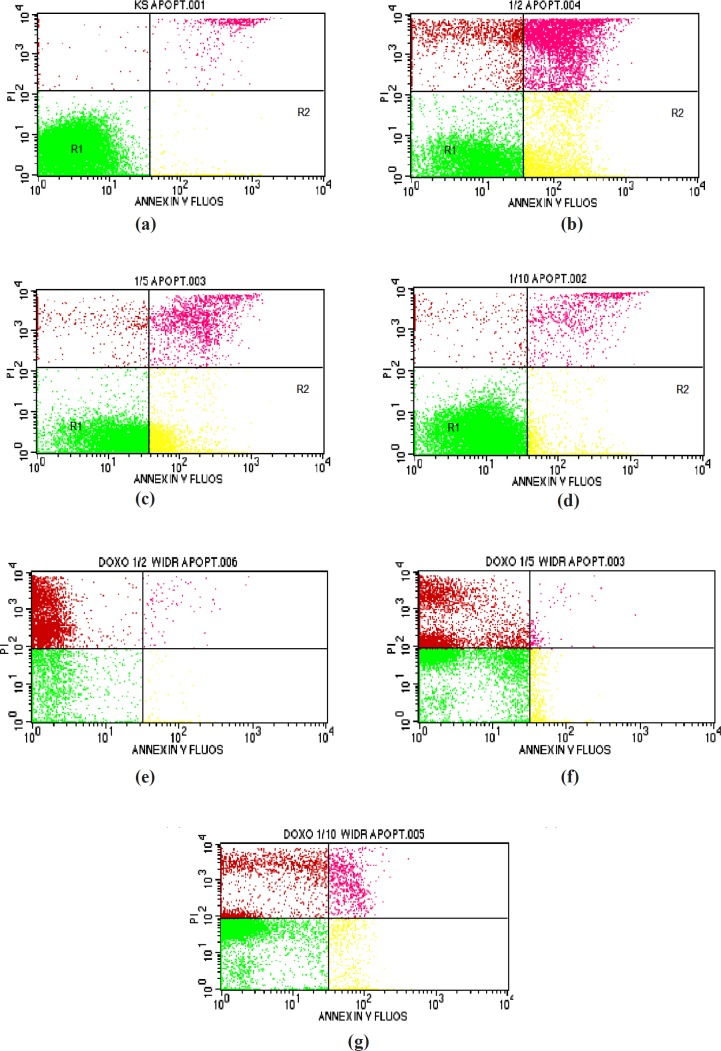
Results of apoptosis analysis in WiDr cells after treatment with various concentration of polyisoprenoid (PI) extract from *A. alba *leaves and doxorubicin (1/2, 1/5 and 1/10 IC50): (a) control; (b) PI, 1/2 IC50; (c) PI, 1/5 IC50; (d) PI, 1/10 IC50; (e) doxorubicin, 1/2 IC50; (f) doxorubicin, 1/5 IC50; and (g) doxorubicin, 1/10 IC50

**Figure 3:**
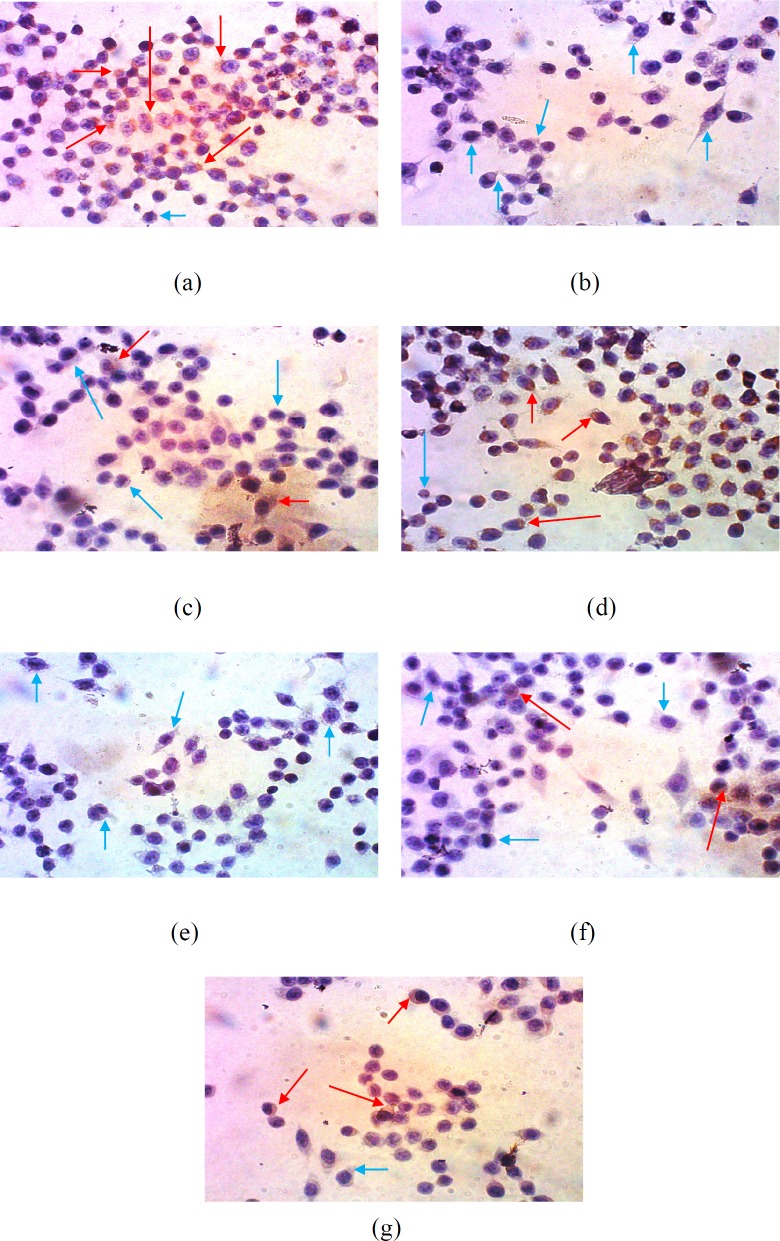
Suppression of COX-2 expression in WiDr cells after treatment with various concentrations of polyisoprenoid (PI) extract from *A. alba *leaves and doxorubicin (1/2, 1/5 and 1/10 IC50): (a) control; (b) PI, 1/2 IC50; (c) PI, 1/5 IC50; (d) PI, 1/10 IC50; (e) doxorubicin, 1/2 IC50; (f) doxorubicin, 1/5 IC50; and (g) doxorubicin, 1/10 IC50 (10 × 40 magnification)

→Positive expression; →Negative expression.

*Cell cycle inhibition analysis*

The effect of PAL on the cell cycle of WiDr cells was analysed using flow cytometry. The DNA content inside the cell was distributed as the G0/G1, S or G2/M phase of the cell cycle. As shown in Table 2 and Figure 1, the percentages of G0-G1 phase profiles for WiDr cells treated with polyisoprenoid extract at 1/2, 1/5, and 1/10-fold of IC_50_ were 62.62, 66.10, and 70.01%, respectively. Meanwhile, the accumulation rates of cells in S phase after treatment with each concentration of PAL were 17.72, 20.35 and 16.46%, respectively. The percentages of control cells in G0-G1 and S phase were 72.20% and 15.83%, respectively. These results indicated that PAL inhibit the growth of cells in the G0-G1 phase. Cessation of the cell cycle at the G0-Gl phase allows the promotion of apoptosis. Cessation of the cell cycle in the Gl phase will provide an opportunity to the damaged cells to be recognized and continue the process of apoptosis (31).

Polyisoprenoids are abundantly found in mangrove plants (13, 18). A previous study has reported that mangrove species, *Lumnitzera racemosa* (*L. racemosa*) had a potent cytotoxic activity against HL-60 leukemia cells line (32). In this context, *L. racemosa* leaves had both presence of longer polyprenols and dolichols (C_60_–C_140_) (18). It has been known that carotenoid is a class of compound with polyisoprenoids structure. The carotenoid compound was able to induce the cell cycle arrest of adenocarcinoma colon cancer cells (COLO 320 HSR, WiDr, and LS174 cell lines) (33, 34). In addition, carotenoid has shown to arrest HL-60 leukemia cells in the G1 phase as well as arrests the cell cycle in different phases and increases apoptosis of MCF-7 cells line (34, 35).

*Apoptosis analysis*

Flow cytometry was performed to determine whether treated cells were undergoing apoptosis following cell cycle arrest. This method was used to calculate viable, necroticand apoptotic cells rapidly. During apoptosis, phosphatidylserine (PS) becomes exposed on the outer cell membrane, and Annexin-V will specifically bind to PS. DNA in damaged cells derived from both necrosis and apoptosis can be stained by propidium iodide, which produces orange to red fluorescence. As it passes through the laser beam, the cells are excited and scatter light to produce fluorescence (36, 37).

As presented in Table 3 and Figure 2, the percentage values of cells undergoing early apoptosis (R2) after treatment with the extract at 1/2, 1/5, and 1/10-fold of IC_50_ were 25.85, 32.66, and 6.44%, respectively. On the other hand, the percentage values of cells undergoing late apoptotic/early necrosis (R3) and late necrosis (R4) were 22.85, 7.66, and 3.76% and 9.22, 2.15, and 1.62%, respectively. The PAL demonstrated positive activity in apoptosis using Annexin-V.

The principle of Annexin-V labelling is the staining of PSon the outer cell membrane. Early apoptotic cells express PS on the outer plasma membrane. PS can be stained by Annexin-V labelling. The cells undergoing late apoptotic and necrosis will lose the integrity of the cell membrane and become permeable to an Annexin-V dye (38).

The mechanism of action of the extract in inducing apoptosis might occur in the early apoptosis phase. The presence of several active compounds, including isoprenoid and polyisoprenoid, were probably responsible for the activity. It was reported that steroid and triterpenoid have anticancer activity by blocking nuclear factor-kappa B, inducing apoptosis, activating transcription, and angiogenesis (39, 40). 

*Suppression of COX-2 expression*

Suppression of COX-2 expression analysis was performed by immunocytochemistry. Expression of COX-2 was qualitatively analysed using a light microscope. The dark/brown colour was indicated as cells that expressed a specific protein, while the cells not expressing any specific protein remained purple/blue in colour (41).

This result is presented in Figure 3 and showed that the number of cells expressing COX-2 was significantly reduced after treatment with 1/2 of IC_50_ of the extract. Meanwhile, the treatment of cells with the extract at 1/5-fold of IC_50_ increased the expression of COX-2. However, compared with the control, this concentration still exhibited inhibition activity towards the expression of COX-2. This result indicated that PAL enabled the inhibition of the expression of COX-2 in WiDr cells in a concentration-dependent manner.

COX-2 is an enzyme that induces inflammation and is highly expressed in tumour cells (42, 43).This enzyme plays an important role in the formation of prostaglandins because it triggers the expression of vascular endothelial growth factor (VEGF). Suppression of VEGF expression will inhibit the formation of new blood vessels used in the migration of cells (metastasis) and cell survival. Therefore, angiogenesis inhibition leads to the death of cancer cells due to the loss of nutrition and oxygen supply from blood vessels and failure to metastasize to other body parts (44). Suppression of COX-2 expression will reduce the production of PGE prostaglandin, which inhibits the expression of Bcl-2, an anti-apoptotic gene (45).

A previous study described that the *in-vitro *haemolytic and anti-inflammatory activities of the ethanol extract *A. alba *was performed using RBC membrane stabilization and anti-protein denaturation assays. The *in-vivo *anti-inflammatory activity was evaluated using an acute inflammatory model of carrageenan-induced rat paw oedema using Wistar rats (n = 6), whereas the level of serum nitric oxide (NO) was estimated as a probable mechanism of action. The ethanol extract in different doses (200 and 400 mg/kg, p.o.) exhibited dose-dependent and significant anti-inflammatory activity *in-vitro *and in an acute model of inflammation (46).

## Conclusion

PAL exhibited anticancer activity against WiDr colon cancer cells. This extract had a mechanism of inhibition of cell cycle at the G0-G1 phase and enables the suppression of COX-2 expression in WiDr cells. This finding might emphasize the potency of polyisoprenoids extract as an anticancer agent against WiDr colon cancer cells. Nevertheless, further investigation on the detail mechanism of the anticancer activity especially *in-vivo* studies is still needed to confirm the *in-vitro* finding of PAL.
